# Profiles of Working Moms’ Daily Time Use: Exploring Their Impact on Leisure

**DOI:** 10.3390/ijerph18052305

**Published:** 2021-02-26

**Authors:** Youngseo Kim, Sehee Hong

**Affiliations:** Department of Education, Korea University, Seoul 02841, Korea; youngsir_kim@naver.com

**Keywords:** working mom, daily time use, latent profile analysis, leisure satisfaction, leisure time

## Abstract

This paper identified latent profiles depending on the patterns of daily time usage amongst working moms in Korea and tested their relations to family- and work-related characteristics. The consequent differences in the levels of leisure attributes were further investigated. Taking a holistic approach, latent profile analysis, one of the person-centered methods, was conducted using data drawn from the seventh year of the Korean Longitudinal Survey of Women and Families (KLoWF), with a sample of 1074 women. The results of this study indicate that three different subtypes of individuals emerged: a low-level care group (82.1%), a medium-level care group (13.8%), and a high-level care group (4.1%). The factors determining the classification for the profiles were the existence of preschool children, household income, gender role attitudes, and domestic help. Work-related factors proved to have no significant effect on time-use patterns. Profile membership was related to leisure attributes as perceived by working moms: the low-level care group reported the highest level of leisure time adequacy and leisure satisfaction, while the high-level care group presented the lowest level of sufficiency and satisfaction in their leisure time. Based on these findings, the article discusses the practical implications for enhancing the quality of life of working mothers.

## 1. Introduction

Across countries, married working women with children (i.e., *working moms*) typically spend a considerable amount of time in two areas of activity, work and family [1], resulting in leisure time constraints. They often have difficulty maintaining a work–family life balance, while performing their invisible duty to spend time cleaning, cooking, and caring for their children. Nevertheless, there may be different patterns of time utilization over the course of a 24-h day depending on personal choice even though each individual is allotted an equal amount of time. Every woman is responsible for distributing her limited time resource to various activities, taking into account the multiple roles, status, and tasks ascribed to herself. While some women decide to spend most of their time on housework and caring labor, others enjoy leisure activities in a balanced manner with labor. Therefore, a closer look at the trend of daily time use may provide a useful insight regarding individual lifestyles and social aspects reflected in them [2,3]. An analysis of time-use patterns can be meaningful for working moms.

### 1.1. Daily Time Use of Working Moms in Korea

Consequently, many researchers have conducted gender-based comparison studies utilizing time-use statistics with a focus on specific work hours, such as domestic labor, caring labor, or wage labor in Korea [4,5,6,7]. The results have been interpreted through the lens of a time availability perspective, a relative resource perspective, and a gender perspective. The basic idea of the time availability perspective is that time spent on domestic labor is reasonably allocated between spouses based on their external constraints such as market labor and internal constraints such as the number and ages of children or other family demands [8]. Wives and husbands decide their time expenditure on unpaid work considering the different amounts of time available for themselves and their spouses [9]. It is a reasonable decision for a couple to allow the spouse to spend less time on household chores when he or she shoulders longer market labor [10].

In addition, the relative resource perspective views the allocation of domestic labor between wives and husbands as a result of negotiations to pursue the greatest possible wellbeing of couples [11]. The theory presupposes household labor as a task that both spouses do not want to carry out. Hence, couples actively utilize their resources, including paid work, education levels, and income, to reduce the responsibilities for onerous domestic labor. The relativity of these resources would function as each spouse’s power for negotiations regarding household work [12]. For instance, a spouse will have greater bargaining power when he or she engages in paid employment and earns a higher income than the other [13]. In effect, the division of unpaid labor reflects the relative economic power of men and women, thereby achieving the maximum efficiency of them [14].

Lastly, a gender perspective assumes that wives suffer from a double-burden of market labor and domestic labor due to unequal power relations [15,16]. Specifically, different activities are prioritized based on socially imposed gender roles, which affect the decision-making of couples in their time allocation. Hochschild and Machung [16] found that married women, despite the recent rise in women’s social status, dedicated their leisure time to conform to gender-specific social norms that regard household chores and family care as female-prerogative tasks. The gender perspective has proved to be a useful framework in explaining the lack of leisure time for married women in Korea [4].

### 1.2. Influencing Factors for Working Moms’ Daily Time Use

It has been reported that women are generally affected by more diverse factors than men are, including their work patterns, the number of workdays, occupation, income, and the age of their children [4]. As for family characteristics, the bringing up of children under the age of 6 has been perceived to influence the gender inequality of time allocation [7]. Working moms who raise preschoolers often carry out a number of tasks at the same time, such as bringing their children to school, purchasing school supplies, and paying insurance [17]. The heavy load of unpaid work gradually decreases as a child enters primary school [6].

Household income is also closely related to the time resources of women. Income poverty has a trade-off relationship with time poverty in that a low-income class devotes longer time to wage labor so as to escape from poverty but faces increased time poverty [18]. Son [19] reported that a higher amount of gross household income reduced the amount of time spent on domestic work among couples and increased their leisure time in Korea. On the other hand, Kim and Ko [6] revealed that the higher the level of household income, the less the leisure time for working women. Kalenkoski et al. [20] analyzed the 2003–2006 American Time Use Survey data and found no significant correlation between the household income and leisure time of individuals. The husband job status has also been found to change the division of labor among couples [21]. When working moms’ spouses engaged in paid employment, the lack of leisure time they had to themselves was further aggravated [6,22].

Additionally, the division of domestic labor has been explained to have a significant effect on the time use of working moms. Braun et al. [23] reported that a husband’s participation in household chores did not necessarily reduce a wife’s time spent on nonmarket work, but had a positive effect on her market work hours. Therefore, it is predicted that wives will be able to spend more time at work as husbands become more involved in household chores. The perception of gender roles is related to the use of daily time for married women. The gender roles have recently drawn more attention because the time availability theory and the relative resource theory have not been applied equally to all cultures and time periods [24,25]. The more strictly gender roles are defined, the more likely it is that individuals have different role perceptions and attitudes shaped by their gender, even in the same time and space [26]. For instance, the traditional male breadwinner ideology, which presupposes that men should take charge of work-related roles in the labor market and women should be responsible for domestic work, deepens gender time inequality [27]. The motherhood ideology, which argues that mothers are primarily responsible for child-rearing, also subordinates women to the household sector [28,29]. These traditional gender role perceptions reduce women’s autonomy and discretion in decision-making on time distribution [30].

Whether or not couples are supported by paid or unpaid housekeepers is related to the time use of working moms. Strober and Weinberg [31] characterized three major strategies in the ways employed women deal with household chores: exchange, sacrifice, and replacement. Replacement means relieving time pressures through the help of family members other than husbands. Parents or parents-in-law often become the primary contributors to women’s participation in economic activities. Replacement strategies through parents are implemented mainly in domestic labor rather than in care labor [31]. The time poverty rate of women decreased when they substituted the labor of others (e.g., paid help and support from family members) for their own unpaid household work [22].

In terms of work characteristics, a full-time employment status is relevant to the daily time use of working moms. Work hours account for a relatively large portion of the daily routine for full-time workers who live a work-oriented life, spending most of their day at work [32]. Consequently, full-time wage earners often suffer from considerable time constraints in their use of time after work. Dual wage earners with young children are the most time-pressed [33]. In addition, a temporary employment status affects the everyday use of time among working moms. Employment contracts with a limited duration, which do not guarantee a retirement age, might increase job insecurity [7]. Temporary workers are aware of the need for leisure, but it is generally difficult for them to enjoy leisure time because of long working hours. Finally, the benefits of flexible working hours will have a significant impact on time allocation for working mothers. The flexible working arrangement system helps employees to effectively manage the dual burden of work and family life. Thus, it enables the reconciliation between career and commitments to family. Women typically experience less work–life conflict regardless of work hours when their work schedules are adjusted with more temporal flexibility and autonomy [34,35,36].

### 1.3. Leisure Attributes of Working Moms

Leisure has been perceived as an essential indicator for the quality of life among working moms [37,38,39]. It helps to effectively relieve stress caused by daily work in both the office and home, and also helps to establish smooth relationships as a member of society [40]. Nevertheless, a lack of leisure time negatively influences a person’s mental health and weakens individual discretion [41]. Leisure time adequacy reflects the mental and emotional resources that a woman can draw on for her everyday pastimes [42]. Besides the absolute amount of leisure time, several researchers have highlighted the importance of perceived time sufficiency in subjective wellbeing [43,44]. Although perceived time adequacy is associated with actual time investment, it represents the subjective experience of stressors more precisely than the objective facet of time resources [43]. According to the comparison theory, the amount of leisure time is compared with that of others because there is no objective standard against which to judge one’s adequacy of leisure time [45]. This process affects the individually perceived time adequacy for recreational activities.

Leisure satisfaction is defined as positive satisfaction or subjective emotions that a person experiences through leisure activities [46]. In general, the level of leisure satisfaction is positively associated with the amount of leisure time and the frequency of leisure activity [47]. The activity theory also suggests that the more active the participation in free-time activities, the greater the happiness and life satisfaction [48,49]. Since numerous studies have reported a positive relationship between leisure satisfaction and life satisfaction [50,51,52,53,54], measuring whether working mothers are satisfied with their leisure experiences may provide important data for developing strategies or programs to help working mothers lead more fulfilled lives [55].

### 1.4. Methodological Considerations

A key limitation of prior literature on women’s time use is that it only deals with certain subcategories of daily hours allocated to market or nonmarket work. Because the subcategories that comprise 24 h organically interact with one another, research results might be limited if various categories of daily activities are not analyzed together in the composition of days. Another limitation is relevant to methodology. Previous studies have identified the average time spent on different activities in a 24 h period by conducting traditional statistical methods, such as multiple regression analysis, logistic regression analysis, and moderation analysis [56]. These variable-centered approaches explain the relationship among variables to analyze a phenomenon in social sciences, assuming that a research sample is extracted from a large homogeneous population [57]. However, there exists a possibility of producing biased estimates when using variable-oriented methods because they overlook important heterogeneity that is not readily apparent in sample data [58].

To overcome this shortcoming, the present study adopted an approach more holistic than variable-centered approaches in investigating multidimensional relations by conducting latent profile analysis (LPA). As a person-centered method, latent profile analysis enables researchers to discover unobserved subgroups by capturing heterogeneity within and between groups of individuals based on the response patterns in several dimensions [59]. For instance, even though a mean of daily time use for leisure reveals that a whole sample has a moderate amount of leisure time, there may exist a group of women who have enough time for leisure and some who do not. Several researchers have used latent profile analysis in work and family studies, uncovering meaningful differences across individual cases [43,60,61]. Such a method helps to characterize women holistically in terms of the typologies of their behavior [62]. In summary, the current study aimed to identify the latent profiles of married working women with children based on their patterns of daily time use. This article further examines whether women’s household and work characteristics can predict their profile membership and whether there is a significant difference in leisure attributes among the profiles. The following research questions were addressed in the study:

1. How many latent profiles emerge from working moms’ time use, and what are the characteristics of each profile?
2. How do family characteristics and work characteristics predict the likelihood of time-use profile membership?
3. Does time-use profile membership predict the level of leisure attributes (leisure time adequacy and leisure satisfaction) of working moms?

## 2. Methods

### 2.1. Data Sources and Samples

The study utilized data from the seventh wave of the Korean Longitudinal Survey of Women and Families (KLoWF) conducted by the Korean Women’s Development Institute. The nationwide panel survey aims to identify the effects of gender policies in Korea and investigate changes in women’s lives, family structure, and jobs. The KLoWF used a stratified multistage sampling design based on Korean Population and Housing Census data, and computer-assisted personal interviewing was utilized as a method of data gathering. Beginning with its first wave to survey 9997 women aged between 19 and 64 years among 9068 households across the nation, data collection has taken place biennially since 2007. Anyone who completes the purpose-of-use survey can have access to the raw data of the KLoWF through their website. In the current study, the participants were comprised of 1074 married working women who engaged in economic activities and raised their children in the survey implemented in 2018. Table 1 outlines the respondent characteristics.

### 2.2. Measures

The variables selected for this study included latent profile variables, predictor variables, and distal outcome variables. Latent profile analysis was performed based on time-use indicators. The time-use variables of each working mom were used for the classification of latent profiles. The four time variables consisted of work hours, leisure hours, household work hours (e.g., cleaning, laundry, and ironing), and care work hours, which were measured as hours on weekdays.

After the identification of time-use profiles, the following variables were used as predictors to examine how the latent profile membership was affected by independent variables: (1) preschool children, (2) household income, (3) husband employment status, (4) husband participation in domestic labor, (5) gender role attitudes, (6) domestic help, (7) full-time employment status, (8) permanent employment status, and (9) flexible working hours.

Among the family characteristics, preschool children were a dichotomous variable, with 1 denoting that a woman had a child under six years of age. Household income referred to the total amount of money a household earned during the past one year. The gross household income expressed on the thousand Korean Won scale was the sum of the pre-tax income of all the members in each household. The number included earned and business incomes alongside financial and real-estate incomes.

The husband employment status was a dichotomous variable, with unemployment (0) as the reference group. The husband participation in domestic labor was measured using five six-point Likert-type items. The respondents were asked to report the frequency of their husband’s involvement in house chores, such as cleaning, dishwashing, laundry, and cooking, from 1 (not at all) to 6 (almost every time a week). The mean score across the items was used in the analysis. The Cronbach’s Alpha reliability coefficient was 0.922. As for gender role attitudes, women indicated their degrees of agreement with six statements (e.g., “Mothers should make most of the decisions on how to bring up their children” and “It is better for men and women to help their children financially together”) on a scale ranging from 1 (strongly disagree) to 4 (strongly agree). The scores for six items were averaged to create an index of gender role perception. Higher values indicate a more positive attitude towards gender equality. The Cronbach’s Alpha reliability for the construct was 0.734. Domestic help was a dichotomous variable for which 1 meant that a woman was supported by housekeepers or caregivers except for her spouse (e.g., parents, parents-in-law, and paid care workers) and no assistance (0) was the reference group.

Among the work characteristics, full-time employment status was a dummy-coded variable, with 1 denoting that a person worked a minimum number of hours defined by their employer and part-time employment (0) being the reference group. Permanent employment status was measured with one item. The research participants were asked to report whether they had permanent work (i.e., permanent work = 1, and temporary work = 0). As for flexible working hours, the respondents were asked to indicate whether they were allowed to deviate from the traditional working arrangements and customize their work schedules, adjusting to their personal needs. A flexible working arrangement was a dichotomous variable, for which 1 meant working with a flexible schedule and no flexibility (0) was the reference group.

Finally, the perceived adequacy of leisure time and leisure satisfaction were utilized as the distal outcome variables for each latent profile. The perception of leisure time (i.e., leisure time adequacy) was measured with one item. The women responded to the statement “I have had enough leisure time during the past year” on a scale of 1–7 (very inadequate to very adequate). As for leisure satisfaction, the participants reported their overall level of leisure satisfaction by answering a single item on a seven-point scale (strongly dissatisfied to strongly satisfied).

### 2.3. Data Analysis

Latent profile analysis was conducted to group the working moms into homogeneous subgroups with regard to their daily time-use patterns [63]. The Mplus 8 program (StatModel, Los Angeles, CA, USA) was used for the analysis. In behavioral science, latent class analysis and latent profile analysis have become popular methods for classifying individuals with similar characteristics into mutually exclusive and exhaustive groups from observed data. Latent profile analysis is performed when the dependent variables are continuous, analyzing the similarities and differences of the response patterns of respondents based on the multivariate distribution of continuous variables [64], while latent class analysis is applied when the dependent variables consist of categorical variables [65].

To determine the best fitting model, the study continuously increased the number of latent profiles and identified the optimal number of those subgroups by comparing models including two to six profiles. As model selection criteria, entropy, information criteria, and relative fit comparisons have been suggested by researchers. The first criterion is entropy, which indicates the quality of classification. It is computed by the mathematical formula below [66].

(1) $$E_{k}=\;1-\;\frac{\sum_{i}\sum_{k}\left[-{\hat{p}}_{ik}\;ln\left({\hat{p}}_{ik}\right)\right]}{n\;ln\left(K\right)}$$

${\hat{p}}_{ik}$ means the posterior classification probabilities of an individual *i* belonging to the class *k*. *n* indicates the sample size, and *K* denotes the number of latent profiles. The entropy provides the overall accuracy of a latent profile assignment, and the value ranges from 0 to 1. An entropy value equal to 1 reveals that perfect classification is achieved, and a high entropy close to 1 represents high classification quality. An entropy level of 0.8 or higher shows acceptable class separation, and low entropy indicates that the latent profiles are not accurately classified [67].

In addition, information criteria were used to decide the most appropriate number of latent profiles. The Akaike information criterion (AIC), Bayesian information criterion (BIC), and sample-size adjusted BIC (SABIC) are given by

(2) AIC=−2ln(L)+2p

(3) BIC=−2ln(L)+p[ln(n)]

*ln*(*L*) represents the log-likelihood, and *p* indicates the number of parameters. *n* is the sample size. The SABIC can be calculated with n* = (*n* + 2)/24 instead of *n* in the BIC formula. For the three information criteria, the model with the smallest values has a better model fit than the other models [68,69,70]. Nylund et al. [71] recommended using the BIC among the information indices based on their simulation studies of this method when determining the number of latent profiles.

In terms of relative fit comparisons, the adjusted Lo–Mendell–Rubin likelihood ratio test (LMR) and parametric bootstrapped likelihood ratio test (BLRT) were used [72,73]. Both tests compare a (*k-1*)-profile model, which is the null model of the hypothesis, with an alternative *k*-profile model. A statistically significant *p*-value implies that a model with *k* profiles should be selected after rejecting a model with (*k-1*) profiles because the *k*-class model fits the data better (Lo et al., 2001).

To explore the determinants of the latent profiles, a multinomial logistic analysis was conducted taking a 3-step approach with covariates and distal outcomes via the R3STEP option in Mplus. The 3-step approach independently assesses the association between latent profile variables and other predictors [74]. First, the basic model including only indicators for latent profile classification is evaluated to avoid the effects of other auxiliary variables in identifying the optimal number of profiles. Second, the previously derived posterior probability distribution is used to decide the latent profile to which each individual is most likely to belong. The final step is to examine the predictive effects of independent variables on the distribution of the latent profiles, considering a classification error. This study used profile membership as a dependent variable. Household- and work-related characteristics were included as independent variables (i.e., possible predictors). A multinomial logistic analysis allowed us to investigate the relations between covariates and profiles. Next, the DU3STEP method in Mplus was utilized to examine the leisure time sufficiency and leisure satisfaction as distal outcomes of profile membership.

## 3. Results

### 3.1. Descriptive Statistics

Table 2 summarizes the descriptive statistics including the means, standard deviations, and correlations of the variables used in this study. Among the four subcategories of time use, paid employment had the highest mean, followed by nonmarket domestic work, leisure, and care work. The combined total of work-related hours exceeded 10 h, including paid work hours (7.58 h) and unpaid work hours (2.94 h). A working mom, on average, spent about 1.86 h per day on leisure activities during the week and 2.28 h per day on household work.

A correlation matrix of selected measures is presented in Table 3. The highest correlation was found between leisure time adequacy and leisure satisfaction. There was a relatively strong association between paid work hours and full-time employment status. Care work hours were closely related to whether a woman raised a child under six years of age. The time devoted to paid work each day was negatively associated with the time spent on leisure activities, domestic labor, and care labor, respectively.

### 3.2. Latent Profiles of Working Moms’ Daily Time Use

For the identification of the latent profiles, statistical criteria including entropy, information criteria, and relative fit comparisons were used to select the model that fitted the observed data the best (see Table 4).

**Figure 1:**
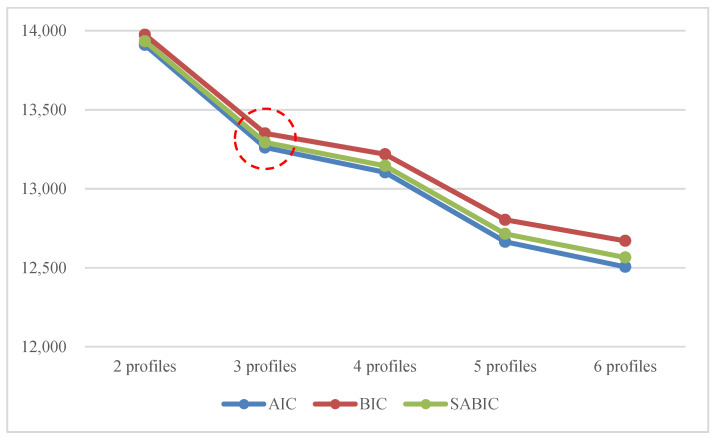
Changes in information indices with increasing profiles.

Models with smaller values of the AIC, BIC, and SABIC are preferred when comparing models with two to six profiles. Figure 1 illustrates the changes in three information indices. Specifically, the AIC, BIC, and SABIC values continuously decreased as the number of latent profiles increased. In this case, the number of profiles can be determined by observing the interval in which the steep slope suddenly changed into a gentle incline in the graph. The model with three latent profiles was superior to any other model, as all three model fit indices showed a sharp decline as illustrated in Figure 1.

The adjusted Lo–Mendell–Rubin likelihood ratio test (LMR-LRT) was not useful in determining the optimal number of latent profiles. It showed a significant *p*-value at a 5% significance level when the number of latent profiles was two or six. No one model was clearly supported based on this criterion. In the BLRT, all the *p*-values were continuously significant from the model with two profiles to that with six profiles. Hence, this test was not used to choose the final LPA model. The entropy was the highest for the three-profile model (0.997), which indicates a more accurate delineation of subgroups. Based on multiple statistical indices, it can be concluded that the three-profile model fits the data best. Although it was difficult to draw a consistent conclusion with the model comparison tests, the information indices became a relatively satisfactory methodological heuristic. Examining the theoretical interpretability of the groups also suggested that the model with three profiles was optimal.

**Figure 2:**
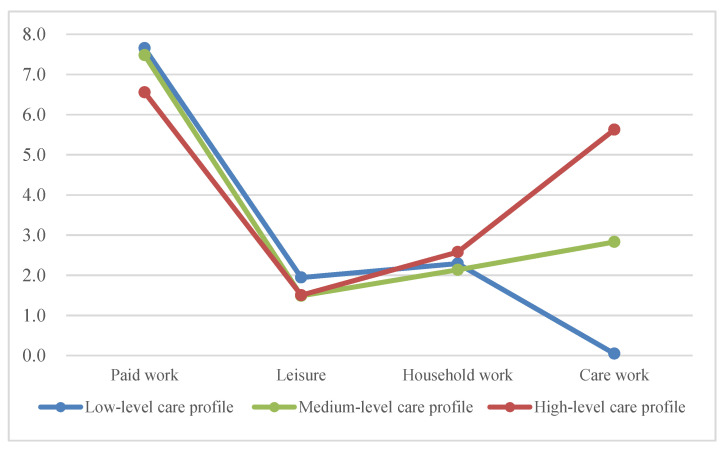
Time-use profiles of working moms.

The latent profiles and their time-use characteristics are presented in Figure 2. Three groups were labeled as follows, accounting for the coefficient values (i.e., means for each time subcategory): a high-level care profile, a medium-level care profile, and a low-level care profile. Overall, all three profiles devoted the largest amount of time to paid work among the four subcategories. Three groups spent approximately similar amounts of time on leisure activities and household tasks, such as washing dishes and cooking.

However, the most noticeable time gap lies in caring for children or the elderly. The first profile is called the “high-level care profile” (n = 44; 4.1% of the sample) because the women in this profile had the highest care labor hours (5.626 h), with relatively high domestic labor (2.578 h) and fewer leisure activities (1.504 h) compared to the other groups. They reported a slightly elevated tendency to reduce leisure time by spending most of their spare time on childcare, even though they spent relatively less amount of time on paid labor than the other two groups. The second profile is called the “medium-level care profile” (n = 148; 13.8% of the sample), characterized by a moderate level of time allocation on all the time-use subscales. The medium-level care profile shows a time-use pattern similar to that of the high-level care profile, while this profile demonstrates a lower amount of time on several subcategories. Furthermore, the amount of leisure time (1.488 h) is lower than other activities in the medium-level care profile. Lastly, the third profile includes the most women and is called the “low-level care profile” (n = 882; 82.1% of the sample). The women in this profile are characterized by the least amount of time on care labor (0.046 h) among the three groups. The profile consists of working moms who report a relatively high amount of leisure time (1.943 h) in comparison to other profiles.

### 3.3. Predicting Time-Use Profile Membership

Multinomial logistic regression was performed with possible predictors of latent profile membership. This analysis examined the extent to which family and work characteristics predict profile membership. The low-level care profile was used as a reference category.

First, the membership likelihood of the low-level care profile was compared to that of the medium-level care profile (See Table 5). Positive significant effects were found for preschool children (*p* < 0.01; OR = 1.351) and household income (*p* < 0.01; OR = 2.953). The odds ratios indicated that belonging to the medium-level care profile was 1.351 and 2.953 times more likely than being a member of the low-level care profile when a woman raised preschool children and when the household income increased by one unit, respectively. Gender role attitudes (*p* < 0.01; OR = 0.485) and domestic help (*p* < 0.001; OR = 0.865) had statistically significant negative effects, meaning that belonging to the medium-level care profile was 0.485 and 0.865 times less likely for every one-unit increase in women’s gender role attitudes and domestic help compared with the low-level care profile.

Next, the comparison between the low-level care profile and the high-level care profile showed that preschool children (*p* < 0.01; OR = 1.467) and household income (*p* < 0.01; OR = 4.078) had significantly positive effects. Women were more likely to belong to the high-level care profile than the low-level care profile when they had to take care of their preschool children and when the household income increased. In addition, negative, significant effects were detected for gender role attitudes (*p* < 0.01; OR = 0.304) and domestic help (*p* < 0.001; OR = 0.819). As indicated by the odds ratios, belonging to the high-level care profile is 0.304 and 0.819 times less likely when the gender role attitude value increases by one unit and when there is someone to assist with household chores except for the spouse. Work characteristics were unrelated to the latent profile membership of the women.

### 3.4. Leisure Time Adequacy and Leisure Satisfaction by Time-Use Profiles

In the final step, the study examined how the distal outcomes (i.e., leisure time adequacy and leisure satisfaction) differed as functions of latent profile. As shown in Table 6, we found significant differences in women’s perceptions of leisure time across the time-use profiles. Women in the low-level care profile perceived significantly higher time adequacy for leisure than the other two profiles. The level of leisure satisfaction also differed across classes. The low-level care profile had the highest level of leisure satisfaction among the three groups, whereas those in the high-level care profile were the least satisfied.

## 4. Discussion

This paper aimed to identify the time-use patterns of working moms in Korea. Employing a person-centered approach, we sought to account for the individual differences in daily time allocation and explore the predictive effects of family and work characteristics on time-use profile membership. Finally, the current study examined how members of latent profiles demonstrated different levels of leisure satisfaction and leisure time adequacy.

### 4.1. Working Moms’ Time-Use Profiles

The overall mean time spent on activities indicated that working moms spent the highest amount of time on paid labor (7.58 h) and the second-highest amount of time on domestic chores (2.28 h). The combined total of the average amount of time spent on work-related activities accounted for more than 10 h a day during the week, causing the limited leisure time available, with an average of 1.86 h per day. Since the average Korean person spends 3.5 h on leisure activities on weekdays and 5.4 h on holidays [75], it is evident that working moms experience time poverty due to relatively longer hours spent on market and nonmarket labor in comparison with men. The gender inequalities in unpaid work translate into a large gender gap in leisure time. This conclusion is in line with prior studies [7,16].

Taking a step further from this, the results of the latent profile analysis reveal that three profiles were identified, which are called the high-level care profile (4.1%), the medium-level care profile (13.8%), and the low-level care profile (82.1%). The medium-level care profile and the high-level care profile spent a higher amount of time on care labor than the overall mean. The three profiles showed stark differences in unpaid care work. Specifically, the average hours per day spent on care activities were about 0.05 h per day during the week for the low-level care profile, while the medium-level care profile spent, on average, about three hours (2.83 h). Surprisingly, leisure time did not increase significantly despite the relatively small amount of time spent on paid work among the high-level care profile. Rather, women who belonged to this profile spent more time on domestic and care labor. As a result, their average duration of care activities was more than five hours a day during the week, which is eight times the amount of time spent on care work by an average working mom (0.66 h). The women in the low-level care profile, however, did not report more leisure time compared to the other profiles, even though they had relatively less care work time than the others. A reduction in working hours does not necessarily ensure longer leisure time. This is because working moms spend their spare time on household chores, care labor, or other necessary tasks after work, not on leisure activities for physical health and wellbeing.

These findings can be explained by the sacrifice strategy [31]. Working moms sacrificed their leisure time to work extra hours to carry out responsibilities for housework and care under the constraints of limited time resources. This study further revealed that working moms selected a sacrifice strategy even for the increased time resources derived from the reduction of working hours. That is, leisure continues to fall behind in the priority of time allocation across the three profiles that emerged. It can be explained that married women generally do not feel entitled to leisure. Rather, they try not to neglect the role of wife and mother at home [76]. Hence, leisure typically ends up being the lowest priority for women. The results can also be interpreted through Henderson [77]’s marginality theory that women who feel marginalized in society often think their lives are at the disposal of other people including their spouses and children. Such a lack of control over life causes women not to place a high priority on their leisure [78]. Accordingly, the reduction of certain work hours does not directly guarantee an increase in leisure time. The relationship between the hours spent on different activities is not simple but complicated for working moms.

### 4.2. Predictive Roles of Family Characteristics and Work Characteristics

Family characteristics were predictive of the latent profile membership of women. Specifically, the existence of preschool children, household income, gender role attitudes, and domestic help had statistically significant effects on the time allocation of working moms. Women with preschool children showed a higher probability of belonging to the high-level or medium-level care profiles than the low-level care profile. This result corresponds to previous research [7,79] in that working moms’ time-use patterns depend on whether they have preschool children or not. Women raising younger children struggle the most to reconcile childcare responsibilities with paid labor. Additionally, the higher the gross household income, the greater the possibility that they become members of the medium-level or high-level care profile instead of the low-level care group. This result contradicts the findings of Son [19], who argues that the higher the household income, the more likely it is that working moms spend more time on leisure activities and less time on domestic tasks. Further research is needed on the relationship between household income and care work hours.

As for gender role perceptions, the more unequal the gender role perception was, the more likely women were to belong to medium-level or high-level care profiles than the low-level care profile. Women’s egalitarian gender role attitudes had a statistically significant effect on the classification for time-use profiles among working moms, disputing the statement of a previous study. Greenstein [80] reported that the gender role attitudes of wives do not influence both spouses’ behavior, while the household division of labor is affected by husbands’ attitudes towards gender equality. The results of this study highlight that women’s initiatives in time management are as important as the absolute amount of time available for leisure activities. It is fundamental to induce an ideological shift of women through gender role resocialization education, public campaigns, and advertisements that help to change their gender role attitudes and behaviors gradually.

Consistent with previous studies [22], women who received no support from home childcare workers were more likely to belong to the medium-level or high-level care profiles than the low-level care profile. This can be interpreted by Strober and Weinberg [31]’s replacement strategy. Domestic help enables double-income couples to have the leeway and time necessary for them to enjoy leisure activities because fewer tasks will be assigned to both spouses at home.

Finally, the nonsignificant relationship between work characteristics and time-use patterns of working moms contradicts previous research regarding full-time employment status [33], permanent employment status [22], and flexible work schedules [34,35,36]. Based on these results, it can be assumed that married working women with children are more profoundly affected by family-related variables such as the division of household chores and the existence of housekeepers other than spouses, than by workplace factors.

### 4.3. Impact on Leisure Outcomes

Even though the total leisure time of the three groups seemed similar in that the difference between the profiles was less than an hour, each profile had significantly different perceptions of leisure time adequacy and different levels of leisure satisfaction. Membership in the low-level care profile led to a better perception of leisure time and a higher level of leisure satisfaction than that of the other profiles. On the contrary, women in the high-level care profile reported a relatively lower level of leisure satisfaction and leisure time sufficiency compared to the others. It is speculated that whether one enjoys quality leisure time without the interruption of others (e.g., children) can be more critical than the absolute amount of leisure time [42]. Several researchers have demonstrated that women’s free time at home is generally not in continuous time blocks [4,81]. The fragmentation of leisure time is particularly intensified by the invasion of the care labor sector, where children’s demands should be frequently accommodated. Working moms often perform leisure activities and nonleisure activities simultaneously (e.g., cooking while listening to the radio and changing their children’s diapers while watching TV), resulting in heightened time pressure, increased stress, and, thus, a decreased quality of leisure [82]. In this case, enjoying leisure as a secondary activity in small units of time may have adverse effects on individual perception of time adequacy. Thus, reducing the heavy burden of care work will allow women to have their own time of relaxation by wholeheartedly concentrating on spare-time activities.

## 5. Limitations and Future Research Suggestions

There are several limitations to this study. The panel data of the Korean Women’s Development Institute enabled us to obtain generalizable results for all working moms in the nation. However, no relationships were detected between work characteristics and the time-use profiles despite a sufficient size of samples. For an in-depth understanding of working moms, future research should include other important environmental variables relevant to work, welfare, or legal systems. Potential predictors might include social policies to promote work–family life balance, such as maternity and paternity leave, and childcare facilities in the workplace [83,84]. Variables related to husbands can also be examined in detail.

Furthermore, the present study only focused on the quantitative interpretation of time-allocation patterns. However, “more time is better” is not necessarily true for working moms. Women who have more free time may become less satisfied with their general leisure experiences due to the fragmentation of leisure time. On the contrary, women who spend longer time on market labor may enjoy leisure activities using their discretion after work. Future research should consider qualitative studies to gain more knowledge of the decision-making process regarding the time utilization among working moms. For example, open-ended and unstructured interviews will be useful for exploring how working moms set priorities and allocate their time differently in everyday life. Finally, this paper conducted a cross-sectional study, but it would be beneficial to conduct longitudinal studies that examine the changes in time-use profiles across longer periods (e.g., latent transition analysis).

## 6. Conclusions

Working moms typically suffer time constraints the most due to their parenting, partner, and personal roles. Although comprehensive insight into women’s time allocation is crucial for offering tailored support to an ever-growing working-mother population, studies in this context are lacking. This study explored the differences in time use between diverse profiles to fill this gap. The results indicate that three time-use profiles emerged, namely, high-level, medium-level, and low-level care profiles. The profile with the lowest leisure time adequacy and the highest dissatisfaction with leisure—the high-level care profile—contained the least number of women. Thus, a substantial number of working moms maintained a proper balance between work and family tasks. This article further investigated the predictive effect of various factors surrounding women, including family and work characteristics. Statistically significant variables influencing the classification for the latent profiles included the existence of preschool children, household income, gender role attitudes, and domestic help. Work-related factors were unrelated to time-use patterns, implying the importance of the family context rather than the workplace context. Finally, leisure attributes differed across time-use profiles. Women who spent less time on their care labor reported higher levels of leisure satisfaction and leisure time sufficiency. These findings suggest that policymakers address the unpaid care work of working women as the first priority to enhance their leisure satisfaction and quality of life.

## Figures and Tables

**Table 1:** Demographic information of the sample (*N* = 1074).

Demographic Variable		Frequency	%
Age	20 to 29 years	4	0.4
	30 to 39 years	259	24.1
	40 to 49 years	696	64.8
	50 to 59 years	107	9.9
	Over 60 years	8	0.8
Education	Less than high school	20	1.9
	High school diploma	433	40.2
	Bachelor’s degree	573	53.3
	Graduate/professional	48	4.5

**Table 2:** Descriptive statistics of variables (*N* = 1074).

Variable			Mean	SD	Min.	Max.
Time use (weekday basis)		Paid work	7.58	1.92	1.00	13.20
		Leisure	1.86	1.09	0.00	6.00
		Household work	2.28	1.04	0.00	8.00
		Care work	0.66	1.46	0.00	10.00
Determinants	Family characteristics	Children under age 6	0.21	0.41	0.00	1.00
		Household income	8.66	0.43	5.70	9.90
		Husband employment status	0.92	0.27	0.00	1.00
		Husband participation in domestic labor	2.52	1.08	1.00	6.00
		Gender role attitudes	2.91	0.36	1.83	4.00
		Domestic help	0.08	0.27	0.00	1.00
	Work characteristics	Full-time employment status	0.78	0.42	0.00	1.00
		Permanent employment status	0.55	0.49	0.00	1.00
		Flexible working hours	0.51	0.50	0.00	1.00
Distal outcome variables		Leisure time adequacy	3.89	1.36	1.00	7.00
		Leisure satisfaction	4.09	1.21	1.00	7.00

Note. Log-transformed income was used for analysis; dummy coding (children = 1 and no children = 0, employed = 1 and unemployed = 0, assistance = 1 and no assistance = 0, full-time employment = 1 and part-time employment = 0, permanent work = 1 and temporary work = 0, flexible = 1 and inflexible = 0).

**Table 3:** Correlation matrix of variables.

Variable	1	2	3	4	5	6	7	8	9	10	11	12	13	14	15
1	1														
2	0.002	1													
3	0.191 **	−0.148 **	1												
4	−0.180 **	−0.123 **	−0.133 **	1											
5	−0.017	0.555 **	−0.122 **	−0.066 *	1										
6	−0.045	0.016	−0.016	0.051	0.013	1									
7	0.078 *	0.043	0.079 **	−0.019	−0.001	0.190 **	1								
8	−0.038	0.151 **	0.034	0.080 **	0.167 **	0.104 **	0.061 *	1							
9	−0.025	0.042	−0.077 *	−0.024	0.029	0.106 **	0.030	−0.023	1						
10	−0.180 **	0.084 **	−0.050	0.042	0.164 **	0.079 **	−0.020	−0.007	0.080 **	1					
11	−0.202 **	−0.059	−0.159 **	0.688 **	−0.010	0.150 **	−0.006	0.120 **	−0.013	0.071 *	1				
12	−0.065 *	0.022	−0.048	0.026	0.061 *	−0.015	−0.026	0.011	0.019	0.044	0.030	1			
13	0.312 *	−0.207	0.056	0.005	−0.070	−0.108	−0.215	−0.234	−0.008	0.164	−0.087	0.018	1		
14	0.032	−0.059	−0.022	−0.015	−0.056	−0.049	−0.008	−0.047	−0.012	−0.040	−0.014	−0.005	−0.195	1	
15	0.039	−0.061 *	0.029	−0.005	−0.054	−0.050	−0.017	−0.051	−0.031	−0.038	−0.006	−0.016	−0.192	0.822 **	1

Note. 1. Household work, 2. Care work, 3. Leisure, 4. Paid work, 5. Children under age 6, 6. Household income, 7. Husband employment status, 8. Husband participation in domestic labor, 9. Gender role attitudes, 10. Domestic help, 11. Full-time employment status, 12. Permanent employment status, 13. Flexible working hours, 14. Leisure time adequacy, 15. Leisure satisfaction. * *p* < 0.05, ** *p* < 0.01.

**Table 4:** Fit indices for models with number of profiles ranging from 2 to 6.

Model	AIC	BIC	SABIC	Entropy	LMR-LRT	BLRT
2 profiles	13,910.400	13,975.129	13,933.838	0.983	*p* < 0.05	*p* < 0.05
3 profiles	13,261.043	13,350.667	13,293.496	0.997	*p* = 0.05	*p* < 0.05
4 profiles	13,103.970	13,218.490	13,145.438	0.941	*p* = 0.42	*p* < 0.05
5 profiles	12,664.282	12,803.698	12,714.764	0.951	*p* = 0.40	*p* < 0.05
6 profiles	12,505.865	12,670.176	12,565.362	0.962	*p* < 0.05	*p* < 0.05

Note. AIC = Akaike information criterion; BIC = Bayesian information criterion; SABIC = sample-size adjusted BIC; LMR-LRT = adjusted Lo–Mendell–Rubin likelihood ratio test; BLRT = bootstrap likelihood ratio.

**Table 5:** Multinomial logistic regression results for predicting profile membership.

Variable		Reference Group: Low-Level Care Profile	
		Medium-Level Care Profile	High-Level Care Profile
		Coef. (*SE*)	Coef. (*SE*)
Family characteristics	Preschool children	0.301 ** (0.107)	0.383 ** (0.146)
	Household income	1.083 ** (0.382)	1.406 ** (0.495)
	Husband employment status	0.358 (0.320)	−1.092 (0.863)
	Husband participation in domestic labor	0.464 (0.291)	0.028 (0.578)
	Gender role attitudes	−0.723 ** (0.275)	−1.192 ** (0.409)
	Domestic help	−0.145 *** (0.030)	−0.200 *** (0.049)
Work characteristics	Full-time employment status	0.591 (0.511)	0.871 (0.908)
	Permanent employment status	0.133 (0.087)	0.136 (0.133)
	Flexible working hours	0.747 (0.422)	−0.094 (0.717)

Note. *SE* = standard error of the coefficient (Coef); dummy coding (children = 1 and no children = 0, employed = 1 and unemployed = 0, assistance = 1 and no assistance = 0, full-time employment = 1 and part-time employment = 0, permanent work = 1 and temporary work = 0, flexible = 1 and inflexible = 0). ** *p* < 0.01, *** *p* < 0.001.

**Table 6:** Leisure attributes by profiles.

Profile	Leisure Time Adequacy		Leisure Satisfaction	
	Mean	Standard Error	Mean	Standard Error
Low-level care profile	3.799	0.050	4.088	0.041
Medium-level care profile	3.622	0.128	4.042	0.099
High-level care profile	3.523	0.240	3.637	0.208
